# Enzyme Catalysis for Sustainable Value Creation Using Renewable Biobased Resources

**DOI:** 10.3390/molecules29235772

**Published:** 2024-12-06

**Authors:** Roland Wohlgemuth

**Affiliations:** 1 Faculty of Chemistry, Lodz University of Technology, Zeromskiego Street 116, 90-924 Lodz, Poland; roland.wohlgemuth.1@p.lodz.pl; 2 Swiss Coordination Committee Biotechnology (SKB), 8021 Zurich, Switzerland; 3 European Society of Applied Biocatalysis (ESAB), 1000 Brussels, Belgium

**Keywords:** biomanufacturing, biocatalysis, enzymes, industrial biotechnology, sustainable chemistry, white biotechnology, green chemistry

## Abstract

Enzyme catalysis was traditionally used by various human cultures to create value long before its basic concepts were uncovered. This was achieved by transforming the raw materials available from natural resources into useful products. Tremendous scientific and technological progress has been made globally in understanding what constitutes an enzyme; what reactions enzymes can catalyze; and how to search, develop, apply, and improve enzymes to make desired products. The useful properties of enzymes as nature’s preferred catalysts, such as their high selectivity, diversity, and adaptability, enable their optimal function, whether in single or multiple reactions. Excellent opportunities for the resource-efficient manufacturing of compounds are provided by the actions of enzymes working in reaction cascades and pathways within the same reaction space, like molecular robots along a production line. Enzyme catalysis plays an increasingly prominent role in industrial innovation and responsible production in various areas, such as green and sustainable chemistry and industrial or white biotechnology. Sources of inspiration include current manufacturing or supply chain challenges, the treasure of natural enzymes, and opportunities to engineer tailor-made enzymes. Making the best use of the power of enzyme catalysis is essential for changing how current products are manufactured; how renewable biobased resources can replace fossil-based resources; and improving the safety, health, and environmental aspects of manufacturing processes to support cleaner and more sustainable production.

## 1. Introduction

Fermentation has been traditionally used in numerous human cultures for creating value by transforming raw materials available from natural resources into useful products, such as fermented food and beverages, as demonstrated, for example, by the isolation and characterization of live yeast cultures in ancient vessels [1]. Bio-archeology tools enable us to study these important human activities that involve enzyme catalysis in value creation. Examples include processing milk into valuable non-perishable milk products like cheese, which are easier to transport [2], or winemaking after the cultivation of grapes [3]. Enzymes were applied in value creation long before the fundamentals of science and the concepts of enzymes, molecules, and money were developed. The word “ferment” was coined by Wilhelm Kühne [4] and was used by German workers. The replacement of the old word ferment with the word enzyme, which was used by English workers [5], has also been discussed in terms of widening the term’s meaning so that it might encompass all catalytic reactions occurring in biological cells. The experimental demonstration by Eduard Buchner in 1897, which showed that whole yeast cells are not needed and a cell-free extract prepared from yeast cells is sufficient for converting D-glucose to ethanol [6], was a breakthrough discovery, for which Eduard Buchner received the Nobel Prize in Chemistry in 1907 as the sole recipient. One hundred years later, Eduard Buchner is considered the father of experimental molecular bioscience [7] due to the tremendous influence of his pioneering experiments in cell-free biocatalysis, which have been key to further advances in enzyme catalysis. The first purification and crystallization of urease in 1926 by James B. Sumner [8] and the subsequent purification and crystallization of pepsin by John H. Northrop [9] were further milestones recognized by Nobel Prizes in Chemistry, demonstrating enzymes to be proteins. These key discoveries inspired the purification and crystallization of a large and increasing number of enzymes, which were classified using the four-digit EC numbers given by the Enzyme Commission (EC) [10]. This enzyme classification system is unique among the classifications of catalysts and enables a modular expansion as novel enzyme classes continue to be discovered.

The importance of enzymes to living organisms in the biosphere as nature’s privileged catalysts and the long history of successful applications of enzyme-catalyzed processes have widely increased interest in the science and technology of enzyme catalysis. The fast growth of structural, functional, mechanistic, and application knowledge concerning enzymes; the tools and technologies available today; and the universe of natural, engineered, and de novo enzymes have elevated enzyme catalysis to the status of a key technology for sustainable value creation in a variety of analytical, synthetic, diagnostic, and therapeutic applications. Enzyme catalysis has been crucial to advancing biochemical analysis, in terms of both the measurement of enzyme activities and the determination of analytes using enzymes in analytical and diagnostic applications [11,12,13]. The fast and precise analysis of nucleic acids has been revolutionized by amplifying DNA in vitro with a thermostable DNA polymerase via polymerase chain reaction (PCR) technology, for which Kary Mullis received a Nobel Prize [14,15]. CRISPR-Cas enzymes are attractive for sensitive and specific nucleic acid detection in the molecular diagnostics of human diseases in point-of-care testing and disease monitoring [16,17]. The inherent chirality and excellent selectivity of enzymes also make them attractive catalysts for preparative applications in organic chemistry [18], asymmetric synthesis [19], green and sustainable chemistry [20,21], and industrial biotransformations [22,23]. The excellent properties of enzymes also have great potential for preparative applications in industrial and white biotechnology [24,25]. The development of the directed evolution of enzymes, for which the 2018 Nobel Prize in Chemistry was awarded to Frances Arnold [26], enabled the engineering and optimization of the properties of enzymes to fit the requirements for enantioselectivity or for certain reaction and process conditions, such as the pH, temperature, or substrate [27,28,29]. Advanced experimental and computational tools have accelerated protein engineering and the development of fit-for-purpose enzymes, which have broadened and increased enzyme applications [30]. Enzyme technologies are essential for innovation in various industrial sectors [31,32,33] and manufacturing environments [34]. Various benefits, such as higher resource efficiency and selectivity, shorter routes, and improved safety, health, environmental, and sustainability aspects, can result from the inclusion of enzyme technologies in novel production processes and the creation of novel value chains starting with renewable resources. In addition to the pharmaceuticals developed and manufactured by using enzymes [35], the development and direct application of therapeutic enzymes have provided tremendous benefits [36].

This work aims to outline the advantageous features of enzyme catalysis, which are important not only for creating economic value through the research, development, and innovation of products but also for resource-efficient and sustainable production processes. Considerations of the type of raw material, the manufacturing route, the process characteristics, and its E-factor, which designates the ratio of waste generated to product [37], are all highly significant for implementing the United Nations Sustainable Development Goals (UN SDGs).

## 2. United Nations Sustainable Development Goals (UN SDGs) and Sustainable Value Creation at Micro and Macro Levels Within Earth System Boundaries

The impact of the activities of a growing human population on various parameters of the Earth system has been increasing since the beginning of the Industrial Revolution. The impact of human activities on the global environment is no longer negligible compared with the impact of natural systems, as in previous periods of history. In contrast, various indicators of anthropogenic influence have changed to such an extent that they have reached the level of natural influence on a global scale. It has even been estimated that we stand at a turning point, where the global mass created by humans exceeds the biomass of all biological organisms living on Earth [38]. These combined anthropogenic effects at the planetary scale have inspired the proposal to define the widely discussed term Anthropocene for a new geological epoch different from the Holocene [39,40,41,42]. A proposal to formally recognize the Anthropocene as an epoch within the geological time scale was introduced by the Anthropocene Working Group [43]. A date in the mid-20th century has been proposed for the onset of the Anthropocene at 12 different sites on five continents [43].

A system approach analyzing the types of boundaries and their status with respect to a safe space for life on Earth [44,45] has attracted significant interest. The need for urgent action is evident, as some planetary boundaries are at a high risk of exceeding safe limits, such as biodiversity loss and the biochemical flows of nitrogen and phosphorus [44,45]. While knowledge of the boundaries and a safe space for life is of fundamental interest in searching for a habitable space and living organisms outside Earth, among the thousands of exoplanets discovered in the universe [46,47], the resolution on the 2030 Agenda for Sustainable Development and the associated UN Sustainable Development Goals (SDGs) adopted by the United Nations General Assembly on 25 September 2015 [48] address life and development on Earth. Since its launch in 2015 until now, the 2030 Agenda for Sustainable Development has already reached its midterm, but progress toward many SDGs requires acceleration, course correction, and a sense of urgency according to the Global Sustainable Development Report 2023 [49].

Demands are increasing for energy, materials, and products satisfying the needs of a growing human population, while the amount of waste accumulated along its production chain and from its consumption is also increasing. It is, however, not only the amount but also the type and chemical composition of the waste that require attention to balance global cycles. With the increasing amount and diversity of materials and products, there will also be a parallel increase in the interactions, not only of the products and materials but also of the related waste, with the biosphere. Therefore, minimizing adverse impacts caused by chemicals and waste requires urgent actions, as the size of the global chemical industry in 2017 already exceeded USD 5 trillion. This has been expected to double by 2023 due to emerging economies. This was outlined in the Global Chemicals Outlook II launched in 2019 in Geneva before the reality of various crises, such as the COVID-19 pandemic [50].

Multiple crises at micro and macro levels are, however, nothing new and have occurred throughout human history at numerous locations. Multiple interconnected crises that have reached global dimensions have been outlined as megathreats which need to be addressed and require action [51]. The various actions required to ensure a habitable planet and provide transitional paths to overcome the multiple crises can be guided by planetary boundaries [44,45]. Resource efficiency, recycling, re-utilization, and damage repair, which are hallmarks of living biological organisms, can also inspire the use of enzymes in creating sustainable value. Empowering human ingenuity and investing in scientific research and innovation can lead to future breakthroughs, more responsible production and sustainable value chains, and new economies.

The raw material resources on Earth today are large but not unlimited, with the amount and distribution of elements varying substantially from very rare to highly abundant elements. The recycling and re-utilization of elements is therefore not only of interest for very rare elements but also for the abundant elements and their global cycles. In the case of carbon, carbon-based resources can consist of fossil resources formed over long geological times, such as coal, gas, and oil, which are biobased resources that are formed on the much shorter timescale of biological organisms, such as microbes, algae, and plants, in addition to carbon waste and greenhouse gas emissions produced by anthropogenic activities or natural biodegradation processes. As carbon is a central element not only for life on Earth but also for all kinds of products needed by mankind, decarbonization on a planetary scale is not possible, and the recycling and re-utilization of greenhouse gas emissions and carbon waste are needed to close the carbon cycle [52].

The utilization of the available materials and energy resources in the biotransformations of biological organisms, their interactions, and metabolic processes represent key functions of the global ecosystems on Earth. More than two decades after the publication of the estimated value of the ecosystem services on our planet [53], it has been suggested that these substantial contributions should be at the core of a new economic paradigm in the Anthropocene [54].

**Figure 1:**
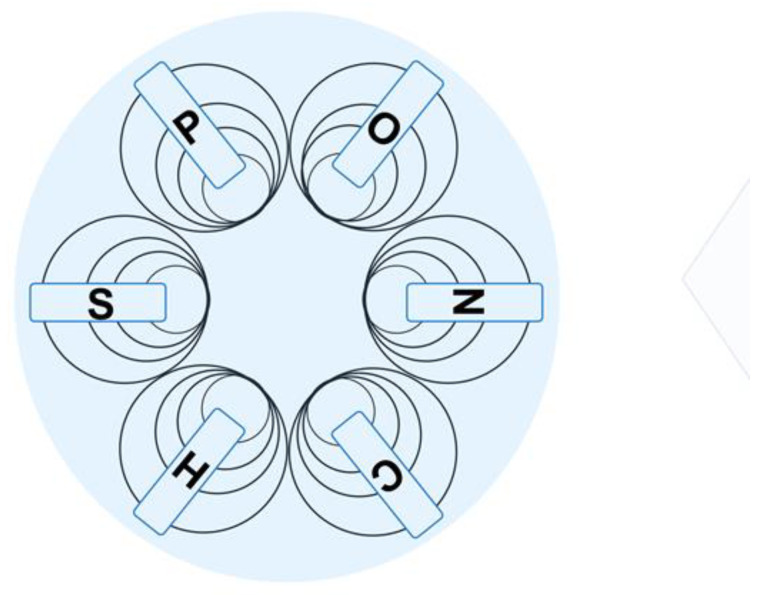
Cycles from micro to macro levels, illustrated by circles of different sizes, of the six key chemical elements—carbon (C), hydrogen (H), oxygen (O), phosphorus (P), nitrogen (N), and sulfur (S)—that occur at high levels in all living organisms.

The microbial world therefore plays not only important roles in creating value at micro levels for higher organisms like plants, animals, and humans but also at macro levels for driving biogeochemical cycles [55]. At the core of the value-creating transformations are enzyme-catalyzed reactions, which constitute the architecture of metabolic processes. It is therefore of much interest to understand their characteristics, evolution, involvement, and roles in global and local cycles of chemical elements, such as the carbon, oxygen, phosphorus, nitrogen, and sulfur cycles [56,57,58,59,60]. Enzymes play important roles in these cycles of chemical elements from micro to macro levels, which is shown schematically in Figure 1 by circles of different sizes representing the six key chemical elements—carbon (C), hydrogen (H), oxygen (O), phosphorus (P), nitrogen (N), and sulfur (S)—that occur at high levels in all living organisms.

The roles of other chemical elements of the periodic system present in the environment are also important, from the molecular level of enzyme-catalyzed reactions and the cellular level of living organisms to their interactions in ecosystems and evolution [61]. Besides the key non-metal elements that occur at high levels in all living organisms, other non-metal chemical elements such as halogens, silicon, and boron and metal chemical elements such as calcium, potassium, sodium, magnesium, zinc, and transition metals are present at lower levels [62,63]. As these and other elements crucial to life need to be efficiently captured, utilized, and stored, as well as released and re-utilized again when needed, the molecular and engineering principles of the underlying biotransformations, as part of the biosynthesis and biodegradation pathways utilized by nature, can serve as highly valuable blueprints from nature for building a sustainable bioeconomy. Inspiration can be provided for new value creation architectures for developing safe and sustainable processes from the beginning, for changing process design from linear to circular processes, and for transitioning toward renewable biobased resources [64]. The choice of biobased raw materials, which can originate from one of the traditional carbon dioxide utilization pathways or novel enzymatic pathways for using carbon dioxide waste from burning fossil resources, can contribute to negative carbon dioxide emissions.

## 3. Enzyme Catalysis for Sustainable Value Creation Using Renewable Biobased Resources

**Figure 2:**
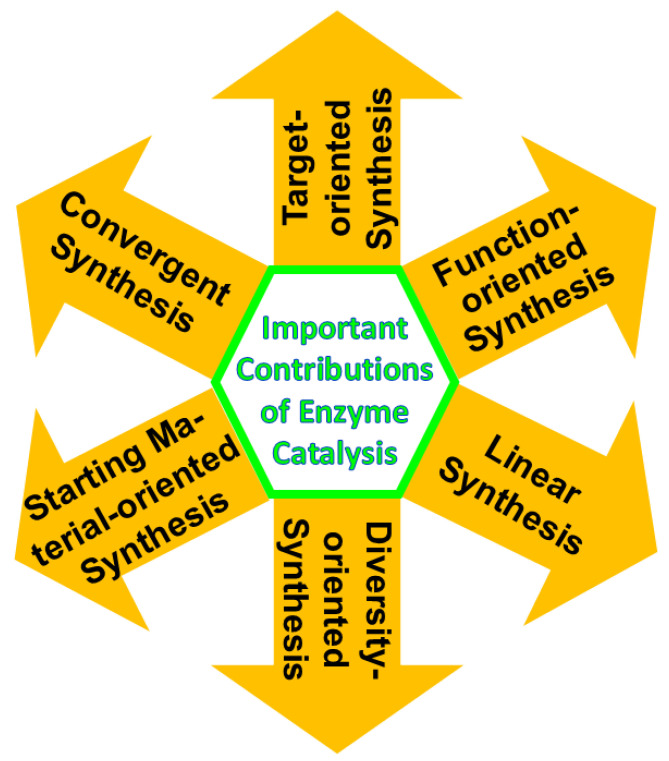
Different concepts of synthesis to which important contributions have already been made and can be made in the future via enzyme catalysis.

The power and selectivity of enzyme catalysis are not only key to resource-efficient biological processes in healthy living organisms on Earth but have also been driving forces and incentives in different areas of synthesis and degradation for developing better processes that provide economic advantages and benefits to health, safety, and the environment [18,19,20,21,22,23,24,25,65]. In the synthetic direction, enzyme catalysis can, both in nature as well as in research, development, and innovation, contribute and create sustainable value for a variety of goals and concepts of synthesis, as illustrated in Figure 2 [66], such as target-oriented synthesis [18,65], linear synthesis [67], convergent synthesis [68], diversity-oriented synthesis [69], and starting material-oriented synthesis [70].

### 3.1. Enzyme Catalysis for Synthetic Applications

Enzyme catalysis in target-oriented synthesis can be guided by comprehensive retrosynthetic analysis and considerations. This involves selecting an adequate synthetic strategy with an optimal sequence of reaction steps and developing suitable chemo-, regio-, and stereoselective enzymes that catalyze the formation of covalent bonds in easily available starting materials to synthesize the desired target molecules of higher value [71,72,73,74]. Synthetic routes can be shortened by using enzyme-catalyzed reaction steps such as the installation and removal of directing or protecting groups, which can be avoided, and the number of reaction steps being decreased [75]. Further advantages can be achieved by coupling two or more enzyme-catalyzed reactions without isolating and purifying the intermediates [76].

Enzyme catalysis can also be valuable in diversity-oriented synthesis for increasing complexity from simple starting materials and expanding the chemical space with a range of diverse molecular structures when generating new leads in drug discovery [77]. Analogs, derivatives, and libraries of natural products can be obtained via the enzymatic diversification of natural products, involving combinatorial biosynthesis, precursor-directed biosynthesis, mutasynthesis, and metabolic pathway engineering strategies, for example, in natural product glycodiversification [78]. The diversity-oriented biosynthesis of numerous new rapamycin-like polyketide molecules has been achieved in a single experiment via accelerated evolution and rapid recombination with a high frequency of polyketide synthase gene clusters [79]. Diverse cyclopropane-containing building blocks have been synthesized using a chemoenzymatic approach, whereby an enzymatic carbene transfer reaction catalyzed by an engineered nitric oxide dioxygenase was followed by subsequent Suzuki–Miyaura cross-coupling with diverse coupling partners [80].

**Figure 3:**
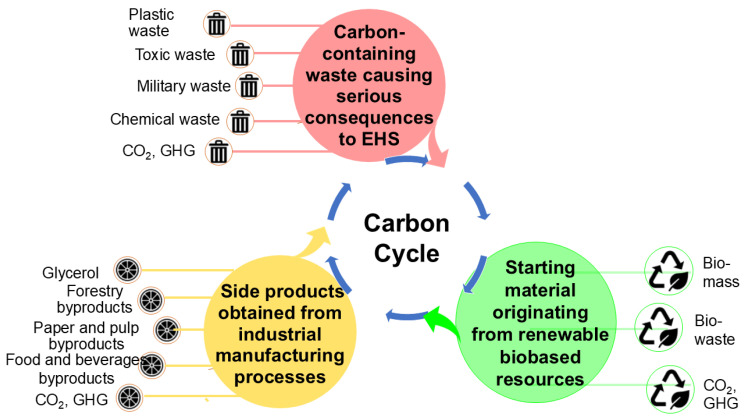
Starting material-oriented synthesis aimed at closing the carbon cycle.

The concept of starting material-oriented synthesis is of much interest when a specific starting material is inexpensive, regionally and reliably available in large amounts, and for transitioning starting materials originating from the extraction of non-renewable resources to the continuous and sustainable supply from resources to close the cycle of key chemical elements. In the case of closing the carbon cycle [52], this can involve, as shown in Figure 3, various types of starting materials, such as (a) waste causing serious consequences for the environment and health, such as plastic and carbon dioxide; (b) a specific side product obtained together with the main product in industrial manufacturing; or (c) a starting material originating from renewable biobased resources [70]. The attention to all the starting materials mentioned above and the tremendous capabilities and the power of enzyme catalysis contribute to solving the challenges of closing the carbon cycle.

### 3.2. Enzyme Catalysis for Closing the Carbon Cycle

Enzyme catalysis has been essential in the history of the carbon dioxide content of our planetary atmosphere, and ribulose-1,5-bisphosphate carboxylase/oxygenase (RubisCO) has evolved into a key enzyme in the global carbon cycle, catalyzing not only the conversion of inorganic carbon into biomass but also providing the oxygen needed for aerobic life [81]. Improved or novel RubisCO enzymes and other natural or engineered enzymes are therefore attractive for catalyzing in vivo the conversion of carbon dioxide to biomass or different biochemical building blocks using the THETA cycle [82]. Exploring completely novel synthetic enzyme systems is of major interest for carbon recycling [83] and catalyzing in vitro the conversion of carbon dioxide to valuable biochemical products, such as glycolate [84], the carboxylation of glycolate to (*R*)-glycerate [85], the use of the CETCH cycle for malate [86] or 6-deoxyerythronolide B [87], and the chemoenzymatic conversion of carbon dioxide and hydrogen to starch [88].

The discovery of synthetic polymers more than a century ago led to an ever-increasing consumption and large-scale production of high-performance materials and their accumulation as waste. Thus, many challenges must be addressed and overcome using new approaches and bold changes to close the carbon cycle and avoid undermining the UN Sustainable Development Goals [89]. One challenge to which enzyme catalysis can contribute to is using synthetic polymer waste as a starting material, which requires a type-specific polymer collection to make them suitable as starting materials. The power of enzyme catalysis provides great opportunities to develop suitable bioprocesses using novel enzymes to catalyze the depolymerization into the corresponding building blocks, thus contributing to a change in the perception of the corresponding synthetic polymers from waste to suitable starting materials [90]. Highly efficient depolymerization of poly(ethylene terephthalate) (PET) into its monomers was developed using an engineered PET hydrolase at 3 mg of enzyme per gram of PET, whereby a space–time yield of 16.7 g L^−1^ h^−1^ was achieved for terephthalate [91]. It is of much interest to expand this result by using PET as a starting material for other synthetic polymers, such as polyamides and polyurethanes. This can be achieved by searching for and engineering suitable depolymerizing enzymes, such as nylonases [92] and urethanases [93], to catalyze the depolymerization of nylon PA6 and polyurethan, respectively.

Another potential material stream for closing the carbon cycle is utilizing specific side products obtained together with the main product in industrial manufacturing as starting materials instead of wasting them. The large surplus of annual glycerol production, which results from the difference between the much larger amount of glycerol generated as a byproduct in biofuel manufacturing and the demand for glycerol, makes glycerol an attractive starting material for sustainable conversion. This can be achieved via enzyme catalysis for various value-added products, such as acrolein; 1,3-dihydroxyacetone; 1,3-propanediol; propionic acid; 3-hydroxypropionic acid; D- and L-lactic acid; mono-, di-, and triacylglycerols; L-serine; and L-tyrosine [94,95,96]. The key advantage of the inherent stereoselectivity of enzyme catalysis is that it enables the enantioselective desymmetrization of glycerol and the synthesis of enantiomerically pure compounds. (*R*)-α-monobenzoate glycerol was obtained in 99% enantiomeric excess via enantioselective glycerol esterification with benzoic acid in 1,4-dioxane using lipase B from *Candida antarctica* immobilized on silica [97]. The high enantioselectivity of glycerol dehydrogenase from *Gluconobacter oxydans* was used in the reductive direction for synthesizing L-glyceraldehyde [98,99]. In screening acetic acid bacteria for microbial glycerol oxidation to D-glycerate, an *Acetobacter tropicalis* strain was identified, which produced 101.8 g L^−1^ D-glycerate in 99% enantiomeric excess [100]. In comparison, glycerol oxidation catalyzed by an evolved alditol oxidase yielded 30 g L^−1^ D-glycerate [101]. Enantioselective glycerol desymmetrization by phosphorylation was demonstrated years ago by preparing L-glycerol-3-phosphate using glycerol kinase, ATP, and the enzymatic regeneration of ATP [102].

### 3.3. Enzyme Catalysis in the Industry

Enzyme catalysis is highly valuable not only for improving the manufacturing processes by using isolated enzymes, cell-free systems, or whole cells for catalyzing the transformation of raw materials to products but also for the preceding manufacturing processes of the starting materials from renewable biobased resources, which are strategically preferred carbon sources for circular processes with net zero carbon emissions [64,103,104,105]. The synthetic routes to products from the biogenic carbon origin of starting materials can not only inspire the design of completely novel products with desired properties but can also address climate, health, safety, and environmental issues and support the complex transition from fossil-based raw materials used in numerous industries to starting materials originating from renewable biobased resources [106,107,108]. Manufacturing processes using enzyme catalysis in microbial cell factories and biobased starting materials have been attractive in the chemical industry for a long time. The early application of a regioselective microbial D-sorbitol oxidation at C5 to L-sorbose in the sequence of reactions for the synthesis of L-ascorbic acid from readily available biobased starting materials enabled the demonstration of its identity with natural vitamin C [109,110]. This has been a key reaction for vitamin C production for decades [111]. The benefits of using biobased starting materials and the application of more and more enzymatic reaction steps in vitamin production [112] have been clearly demonstrated. The milestone of vitamin B_12_ production from D-glucose using recombinant strains expressing highly productive biosynthetic enzymes demonstrates that the greatest synthetic challenges can be solved by characterizing the biosynthetic enzymes and utilizing them (a) in a cell-free system for vitamin B_12_ synthesis [113,114] or (b) in whole-cell systems for developing an industrial process to make the global vitamin B_12_ supply possible [115]. Carefully controlled enzyme catalysis in microbial cell factories can replace lengthy chemical synthesis [116], facilitate and widen synthetic access to natural products [117], and it is becoming more important in the chemical industry for replacing fossil-derived chemicals with biobased chemicals [118,119]. The diverse composition of underutilized carbon sources such as agricultural, forestry, food, and other residues can thereby provide a selection of suitable biobased raw materials for which improved feedstock utilization can be developed to manufacture a broad range of valuable biobased chemicals [120,121,122]. Enzyme catalysis can thereby reduce complexity and simplify manufacturing processes [123], but whether a particular synthetic challenge is best addressed by using enzymes in whole-cell or cell-free forms depends on factors such as the involved enzyme types, substrates, and products or the research stage of screening, process development, or production.

Key factors influencing the economic success and viability of a bioprocess from a biobased raw material to a biobased chemical and its competitiveness with the corresponding fossil-derived chemical are the product value, process metrics, process complexity, quality, application, and customer benefits.

**Figure 4:**
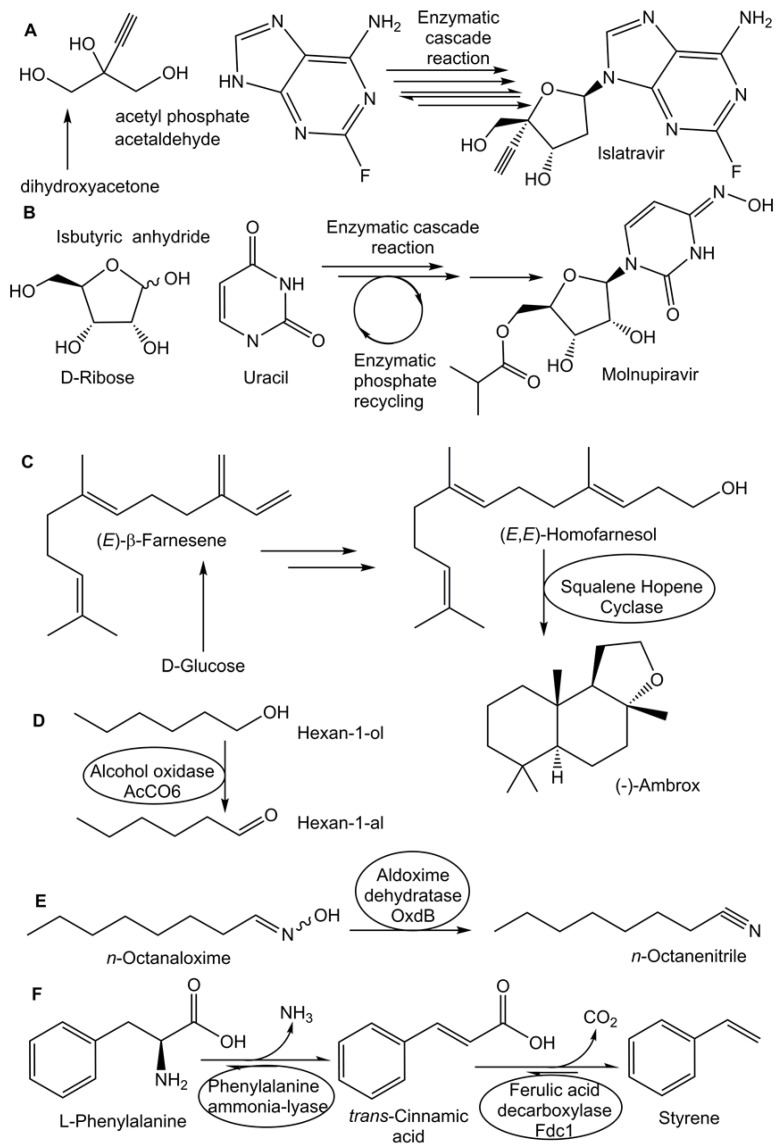
Selected enzyme-catalyzed reactions to valuable biochemical products utilizing starting materials from renewable biobased resources. The enzymatic synthesis of the antiviral islatravir from dihydroxyacetone has been achieved in a reaction cascade (**A**) using five engineered enzymes and four auxiliary enzymes [129,130,131]. The efficient enzymatic synthesis of molnupiravir from biobased D-ribose and uracil (**B**) in three steps enabled rapid manufacturing of the antiviral agent molnupiravir [132] at a large scale. Complete conversion has been achieved in the squalene hopene cyclase-catalyzed reaction (**C**) of (*E,E*)-homofarnesol, which was obtained from biobased (*E*)-β-farnesene produced by fermentation, to (-)-ambrox (**C**) at up to 300 g L^−1^ substrate concentration [140,141]. Hexan-1-al was obtained by the alcohol oxidase-catalyzed oxidation (**D**) of hexan-1-ol [142]. Aldoxime dehydratase-catalyzed water elimination from *n*-octanaloxime was converted to *n*-octanenitrile (**E**) at extremely high substrate concentrations in 24 h [148,149]. Styrene was produced from L-phenylalanine via *trans*-cinnamate (**F**) in a two-step whole-cell biotransformation [147].

Biobased starting materials and enzyme catalysis have become well established in manufacturing high-quality pharmaceuticals and pharmaceutical intermediates [20,23,25,71,75,124,125,126]. An environmental impact analysis of pharmaceutical processes according to non-renewable and renewable feedstock types and processing routes has shown the sustainability potential of the rapidly growing global pharmaceutical market [127]. Selected examples of enzyme catalysis using starting materials from biobased resources for the production of pharmaceuticals and biomaterials are provided in the following section. The enzymatic synthesis of β-lactam nuclei from biobased starting materials, side chains, and their enzymatic coupling have, due to their shorter and cleaner routes, replaced traditional chemical routes, leading to more economical and sustainable manufacturing [128]. Enzyme-catalyzed reactions are widespread in the manufacturing of antivirals, such as the enzymatic synthesis of islatravir, a nucleoside analog for investigational HIV treatment [129]. The enzymatic synthesis of islatravir as a single stereoisomer was achieved with 51% overall yield in a reaction cascade using five engineered enzymes and four auxiliary enzymes, whereby biobased starting materials such as dihydroxyacetone [130] and acetyl phosphate were also used (see Figure 4A). Furthermore, the number of reaction steps was shortened compared with previous routes [129]. Further improvements were achieved by inverting the enzymatic oxidation and phosphorylation steps and evolving the galactose oxidase to oxidize the phosphorylated triol, which, together with process development and the improved gas–liquid mass transfer of oxygen, resulted in more sustainable aerobic oxidation with an excellent yield at the multi-kilogram scale [131]. The importance of fast process design is evident when considering the urgency and high demand for antiviral drugs against SARS-CoV-2 [21]. The design and optimization of an efficient and scalable enzymatic synthesis of molnupiravir from easily available biobased D-ribose and uracil (see Figure 4B), which shortened the original chemical synthesis to three steps and improved the overall yield, enabled the rapid, large-scale supply of the antiviral agent molnupiravir [132]. Enzyme catalysis in a novel reaction cascade involving a lipase, engineered ribosyl-1-kinase and uridine phosphorylase, pyruvate oxidase, and acetate kinase for recycling phosphate was key for rapidly reaching the manufacturing scale [132].

Biomaterials used for medical and cosmetic applications can be produced with a good control of high-quality biobased starting materials using a multi-step enzymatic synthesis of well-defined biopolymers. The advantages of selective enzyme catalysis are clearly evident in the precise polymerization, without using protecting groups, of monosaccharides to homopolysaccharides and complex heteropolysaccharides such as glycosaminoglycans [133,134,135]. The coupling of the two enzymatic reaction modules used for synthesizing the two building blocks, UDP-α-*N*-acetyl-D-glucosamine and UDP-D-glucuronic acid from biobased D-glucuronic acid and *N*-acetyl-D-glucosamine, including ATP regeneration, with their controlled hyaluronan synthase-catalyzed polymerization, enabled the synthesis of high-quality hyaluronic acids [136].

Enzyme catalysis has traditionally provided benefits for converting biobased raw materials to food and food ingredients. This is attractive in the preparation of health-promoting food ingredients and metabolites, such as the enzymatic production of prebiotic oligosaccharides [137], bioactive lipids containing omega-3 fatty acids [138], or urolithins [139].

The use of enzyme catalysis also provides great benefits in manufacturing naturally derived fragrance ingredients when all carbon atoms of the biobased starting material end up in the product, as demonstrated in the biomanufacturing of (-)-ambrox via the squalene hopene cyclase-catalyzed bioconversion (see Figure 4C) of (*E,E*)-homofarnesol [140,141]. The starting material (*E,E*)-homofarnesol was thereby obtained from the biobased (*E*)-β-farnesene, which was produced by fermentation [140,141]. Excellent process metrics with a complete conversion of up to 300 g L^−1^ substrate concentration were achieved [141]. An engineered alcohol oxidase enabled the enzymatic synthesis of a broad range of aldehydes (see Figure 4D) via the selective oxidation of the corresponding primary alcohols [142].

Enzyme catalysis has, since its beginnings, been of fundamental importance in producing novel and sustainable approaches for the asymmetric synthesis of valuable chiral compounds [18,19,143], which have found broad applications in various industrial sectors [20,21,22,23,144]. The discovery or engineering of enantiocomplementary enzymes provides the opportunity for synthetic access to both enantiomers of a chiral compound [145]. Enzyme catalysis is also very attractive for the asymmetric isotopic labeling of molecules of biobased or synthetic origin, such as the highly selective single-step asymmetric reductive deuteration using NADH-dependent reductases [146]. Enzyme catalysis using biobased starting materials has become well established for manufacturing high-value chiral compounds, and there has been a continued intensification of enzymatic processes toward medium-value and commodity products. An enzymatic manufacturing strategy used for the fossil-derived commodity chemical styrene, which is used for numerous syntheses of polymers and chemicals, was developed at a laboratory scale by using an efficient, two-step, whole-cell biotransformation from L-phenylalanine via trans-cinnamate to yield a concentration of about 25 g L^−1^ of styrene (see Figure 4F), despite the toxicity of styrene to cells [147].

Excellent conversion and productivity were achieved in synthesizing aliphatic nitriles via aldoxime dehydratase-catalyzed water elimination from aldoximes (see Figure 4E), which can be derived from renewable biobased alcohols [148]. The aldoxime solubility could be increased by 10% (*v*/*v*) ethanol, and extremely high substrate concentrations of *n*-octanaloxime of up to 1.4 kg L^−1^ could be converted to *n*-octanenitrile in 24 h [149]. Although multiple requirements and several criteria need to be met for commodity products to be competitive with traditional routes, the increasing number of biotransformations of biomass to commodity products via the use of enzymes and cells and improvements and strategies in the utilization of waste feedstock look promising for sustainable biomanufacturing [121,150].

These benefits and favorable safety, health, and environmental aspects of enzyme catalysis have made this technology highly attractive for the sustainable manufacturing of products via SDG-fit novel processes from the laboratory scale to the large industrial scale [20,21,22,23,24,25].

## 4. Discussion

The increasing use of biobased resources and enzyme catalysis for manufacturing valuable products in various industrial sectors contributes to achieving more than half a dozen UN Sustainable Development Goals and transformations, which have been introduced as operationalized modules on how to achieve the Sustainable Development Goals [151]. The dimensions of biobased resources and the advances in enzyme catalysis provide hope and support for accelerating these encouraging developments to produce biobased starting materials by industrially viable routes to utilize carbon dioxide over different time scales [106,152]. As many platform chemicals that are used in numerous applications are manufactured from fossil-based resources, it is particularly interesting that the carbon-negative bioproduction of the common solvents acetone and isopropanol with a productivity of about 3 g L^−1^ h^−1^ has been demonstrated at an industrial pilot scale [153].

In order to provide transparency about the origin of the starting materials, a globally harmonized labeling system could support clear communication along the entire value chain and the progress in transitioning from fossil resources. This would also support decision making on using a starting material from carbon dioxide-utilizing biobased or fossil resources. Standardization is also desirable in the description of enzyme catalysis, and the STRENDA guidelines [154], which are recommended by more than 50 journals, provide a global framework for describing experimental enzyme function data and building the STRENDA database [155] as a functional counterpart to the PDB structure database. The standardized description of experimentally verified data on enzyme functions and enzymatic reactions will not only be valuable for further scientific discoveries in sustainable value creation using renewable biobased resources but also for technological innovations in various industrial sectors.

Overall, enzyme catalysis and its applications (see Table 1) have great potential to contribute to the bioeconomy [156,157] and the UN Sustainable Development Goals.

## 5. Conclusions

The scientific and technological advances in enzyme catalysis have led not only to the tremendous growth of fundamental knowledge and understanding of life on our planet but also to a large and rapidly increasing number of applications, to the extent that the present can be considered a golden age for enzyme catalysis. Enzyme-catalyzed processes have transitioned from a niche area to a powerful, highly selective, and sustainable technology, creating value in many applications by providing economic, health, safety, and environmental benefits from biobased resources. The summary of synthetic, industrial, environmental, analytical, diagnostic, and therapeutic applications in Table 1 and the indicated examples demonstrate the transformative power of enzyme catalysis.

As transformations catalyzed by enzymes have contributed to shaping the Earth, the power of enzyme catalysis can also contribute to stabilizing the Anthropocene within planetary boundaries. However, the dimensions of the global challenges in space and time require bold and consistent actions by all stakeholders across multiple scales.

## 6. Future Directions

Whether naturally occurring, modified, or engineered/synthesized de novo, novel enzyme functions that selectively catalyze desirable transformations from renewable biobased resources or directly utilize carbon dioxide are of fundamental interest and deserve bold investments in research, development, and innovation. The entire innovation pipeline from idea to discovery, rapid prototyping, reaction, and process development to intensification and scaling depends on successful cooperation between people from all the involved areas in order to deliver sustainable value in time and at scale.

## Figures and Tables

**Table 1:** Selected examples of enzyme catalysis application areas summarized under various categories of applications.

Summary of Enzyme Catalysis Applications	Examples
Synthetic Applications	Target-oriented synthesis Linear synthesis Convergent synthesis Diversity-oriented synthesis Starting material-oriented synthesis
Environmental Applications	Degradation of carbon-containing waste and depolymerization of plastic waste Conversion of industrial side products and conversion of biowaste Utilization of carbon dioxide
Industrial Applications	Manufacturing of chemicals and biochemicals Manufacturing pharmaceutical intermediates Manufacturing pharmaceuticals Manufacturing flavors and fragrances and manufacturing of food ingredients
Analytical Applications	Enzymatic analysis of metabolites
Diagnostic Applications	Measurements of activities of human enzymes Polymerase chain reaction Enzymatic analysis of human metabolites
Therapeutic Applications	Enzyme replacement therapy Molecular medicine

## Data Availability

No new data were created or analyzed in this study. Data sharing is not applicable to this article.

## References

[1] Aouizerat T., Gutman I., Paz Y., Maeir A.M., Gadot Y., Gelman D., Szitenberg A., Drori E., Pinkus A., Schoemann M. (2019). Isolation and characterization of live yeast cells from ancient vessels as a tool in bio-archaeology. mBio.

[2] Salque M., Bogucki P.I., Pyzel J., Sobkowiak-Tabaka I., Grygiel R., Szmyt M., Evershed R.P. (2013). Earliest evidence for cheese making in the sixth millennium BC in northern Europe. Nature.

[3] McGovern P., Jalabadze M., Batiuk S., Callahan M.P., Smith K.E., Hall G.R., Kvavadze E., Maghradze D., Rusishvili N., Bouby L. (2017). Early Neolithic wine of Georgia in the South Caucasus. Proc. Natl. Acad. Sci. USA.

[4] Kühne W. (1877). Über das Verhalten verschiedener organisirter und sog. ungeformter Fermente. Verhandlungen Naturhistorisch-Med. Ver. Heidelb. Neue Folge.

[5] Teich M. (1981). Ferment or Enzyme: What’s in a name?. Hist. Philos. Life Sci..

[6] Buchner E. (1897). Alkoholische Gährung ohne Hefezellen. Ber. Dt. Chem: Ges..

[7] Jaenicke L. (2007). Centenary of the Award of a Nobel Prize to Eduard Buchner, the Father of Biochemistry in a Test Tube and Thus of Experimental Molecular Bioscience. Angew. Chem. Int. Ed..

[8] Sumner J.B. (1926). The isolation and crystallization of the enzyme urease: Preliminary paper. J. Biol. Chem..

[9] Northrop J.H. (1930). Crystalline pepsin: I. Isolation and tests of purity. J. Gen. Physiol..

[10] McDonald A.G., Tipton K.F. (2023). Enzyme nomenclature and classification: The state of the art. FEBS J..

[11] Bergmeyer H.U. (1983–1986). Methods of Enzymatic Analysis.

[12] Soleimany A.P., Bhatia S.N. (2020). Activity-based diagnostics: An emerging paradigm for disease detection and monitoring. Trends Mol. Med..

[13] Peng P., Liu C., Li Z., Xue Z., Mao P., Hu J., Xu F., Yao C., You M. (2022). Emerging ELISA derived technologies for in vitro diagnostics. Trends Anal. Chem..

[14] Saiki R.K., Gelfand D.H., Stoffel S., Scharf S.J., Higuchi R., Horn G.T., Mullis K.B., Erlich H.A. (1988). Primer-directed enzymatic amplification of DNA with a thermostable DNA polymerase. Science.

[15] Jia H., Guo Y., Zhao W., Wang K. (2014). Long-range PCR in next-generation sequencing: Comparison of six enzymes and evaluation on the MiSeq sequencer. Sci. Rep..

[16] Liu T.Y., Knott G.J., Smock D.C.J., Desmarais J.J., Sungmin Son S., Abdul Bhuiya A., Shrutee Jakhanwal S., Noam Prywes N., Agrawal S., Díaz de León Derby M. (2021). Accelerated RNA detection using tandem CRISPR nucleases. Nat. Chem. Biol..

[17] Kaminski M.M., Abudayyeh O.O., Gootenberg J.S., Zhang F., Collins J.J. (2021). CRISPR-based diagnostics. Nat. Biomed. Eng..

[18] Faber K., Fessner W.D., Turner N.J. (2015). Science of Synthesis: Biocatalysis in Organic Synthesis.

[19] Wohlgemuth R. (2010). Asymmetric biocatalysis with microbial enzymes and cells. Curr. Opin. Microbiol..

[20] Wu S., Snajdrova R., Moore J.C., Baldenius K., Bornscheuer U.T. (2021). Biocatalysis: Enzymatic synthesis for industrial applications. Angew. Chem. Int. Ed..

[21] Alcántara A.R., Domínguez de María P., Littlechild J.A., Schürmann M., Sheldon R.A., Wohlgemuth R. (2022). Biocatalysis as Key to Sustainable Industrial Chemistry. ChemSusChem.

[22] Liese A., Seelbach K., Wandrey C. (2009). Industrial Biotransformations. Second, Completely Revised and Extended Edition.

[23] Ghisalba O., Meyer H.P., Wohlgemuth R. (2009). Industrial biotransformation. Encyclopedia of Industrial Biotechnology: Bioprocess, Bioseparation, and Cell Technology.

[24] Arbige M.V., Shetty J.K., Chotani G.K. (2019). Industrial enzymology: The next chapter. Trends Biotechnol..

[25] Meyer H.P., Eichhorn E., Hanlon S., Lütz S., Schürmann M., Wohlgemuth R., Coppolecchia R. (2013). The use of enzymes in organic synthesis and the life sciences: Perspectives from the Swiss Industrial Biocatalysis Consortium (SIBC). Cat. Sci. Technol..

[26] Arnold F.H. (2019). Innovation by evolution: Bringing new chemistry to life (Nobel Lecture). Angew. Chem. Int. Ed..

[27] Reetz M.T. (2022). Witnessing the birth of directed evolution of stereoselective enzymes as catalysts in organic chemistry. Adv. Synth. Catal..

[28] Bornscheuer U.T. (2017). The fourth wave of biocatalysis is approaching. Phil. Trans. R. Soc. A.

[29] Zeymer C., Hilvert D. (2018). Directed evolution of protein catalysts. Ann. Rev. Biochem..

[30] Buller R., Lutz S., Kazlauskas R.J., Snajdrova R., Moore J.C., Bornscheuer U.T. (2023). From nature to industry: Harnessing enzymes for biocatalysis. Science.

[31] Vogel A., May O. (2019). Industrial Enzyme Applications.

[32] Adams J.P., Brown M.J., Diaz-Rodriguez A., Lloyd R.C., Roiban G.D. (2019). Biocatalysis: A pharma perspective. Adv. Synth. Catal..

[33] Heath R.S., Ruscoe R.E., Turner N.J. (2022). The beauty of biocatalysis: Sustainable synthesis of ingredients in cosmetics. Nat. Prod. Rep..

[34] Hecht K., Meyer H.P., Wohlgemuth R., Buller R. (2020). Biocatalysis in the Swiss manufacturing environment. Catalysts.

[35] Devine P.N., Howard R.M., Kumar R., Thompson M.P., Truppo M.D., Turner N.J. (2018). Extending the application of biocatalysis to meet the challenges of drug development. Nat. Rev. Chem..

[36] Cioni P., Gabellieri E., Campanini B., Bettati S., Raboni S. (2022). Use of Exogenous Enzymes in Human Therapy: Approved Drugs and Potential Applications. Curr. Med. Chem..

[37] Sheldon R.A. (2023). The E factor at 30: A passion for pollution prevention. Green Chem..

[38] Elhacham E., Ben-Uri L., Grozovski J., Bar-On Y.M., Milo R. (2020). Global human-made mass exceeds all living biomass. Nature.

[39] Crutzen P.J. (2002). Geology of Mankind. Nature.

[40] Steffen W., Grinevald J., Crutzen P., McNeill J. (2011). The Anthropocene: Conceptual and historical perspectives. Phil. Trans. R. Soc. A..

[41] Lewis S.L., Maslin M.A. (2015). Defining the Anthropocene. Nature.

[42] Waters C.W., Zalasiewicz J., Summerhayes C., Barnosky A.D., Poirier C., Gałuszka A., Cearreta A., Edgeworth M., Ellis E.C., Ellis M. (2016). The Anthropocene is functionally and stratigraphically distinct from the Holocene. Science.

[43] Waters C.N., Turner S.D., Zalasiewicz J., Head M.J. (2023). Candidate sites and other reference sections for the Global boundary Stratotype Section and Point of the Anthropocene series. Anthr. Rev..

[44] Steffen W., Richardson K., Rockström J., Cornell S.E., Fetzer I., Bennett E.M., Biggs R., Carpenter S.R., De Vries W., De Wit C.A. (2015). Planetary boundaries: Guiding human development on a changing planet. Science.

[45] Richardson K., Steffen W., Lucht W., Bendtsen J., Cornell S.E., Donges J.F., Drüke M., Fetzer I., Bala G., von Bloh W. (2023). Earth beyond six of nine planetary boundaries. Sci. Adv..

[46] Mayor M. (2020). Nobel Lecture: Plurality of worlds in the cosmos: A dream of antiquity, a modern reality of astrophysics. Rev. Mod. Phys..

[47] National Academies of Sciences, Engineering, and Medicine (2023). Pathways to Discovery in Astronomy and Astrophysics for the 2020s.

[48] United Nations General Assembly Seventieth Session, Transforming Our World: The 2030 Agenda for Sustainable Development. 2015, A/RES/70/1, 1–35. https://sdgs.un.org/2030agenda.

[49] United Nations Department of Economic and Social Affairs, Global Sustainable Development Report 2023, Advance, Unedited Version. https://sdgs.un.org/gsdr/gsdr2023.

[50] United Nations Environment Programme (2019). Global Chemicals Outlook II—From Legacies to Innovative Solutions: Implementing the 2030 Agenda for Sustainable Development.

[51] Roubini N. (2022). Megathreats—Ten Dangerous Trends That Imperil Our Future, And How to Survive Them.

[52] Shaw W.J., Kidder M.K., Bare S.R., Delferro M., Morris J.R., Toma F.M., Senanayake S.D., Autrey T., Biddinger E.J., Boettcher S. (2024). A US perspective on closing the carbon cycle to defossilize difficult-to-electrify segments of our economy. Nat. Rev. Chem..

[53] Costanza R., dArge R., de Groot R., Farber S., Grasso M., Hannon B., Limburg K., Naeem S., Oneill R.V., Paruelo J. (1997). The value of the world’s ecosystem services and natural capital. Nature.

[54] Costanza R., de Groot R., Braat L., Kubiszewski I., Fioramonti L., Sutton P., Farber S., Grasso M. (2017). Twenty years of ecosystem services: How far have we come and how far do we still need to go?. Ecosyst. Serv..

[55] Falkowski P.G., Fenchel T., Delong E.F. (2008). The microbial engines that drive Earth’s biogeochemical cycles. Science.

[56] Regnier P., Resplandy L., Najjar R.G., Ciais P. (2022). The land-to-ocean loops of the global carbon cycle. Nature.

[57] Huang J., Liu X., He Y., Shen S., Hou Z., Li S., Li C., Yao L., Huang J. (2021). The oxygen cycle and a habitable Earth. Sci. China Earth Sci..

[58] Reinhard C.T., Planavsky N.J., Gill B.C., Ozaki K., Robbins L.J., Lyons T.W., Fischer W.W., Wang C., Cole D.B., Konhauser K.O. (2017). Evolution of the global phosphorus cycle. Nature.

[59] Zhang X., Ward B.B., Sigman D.M. (2020). Global nitrogen cycle: Critical enzymes, organisms, and processes for nitrogen budgets and dynamics. Chem. Rev..

[60] Sievert S.M., Kiene R.P., Schulz-Vogt H.N. (2007). The sulfur cycle. Oceanography.

[61] Williams R.J.P., Rickaby R.E.M. (2012). Evolution’s Destiny: Co-Evolving Chemistry of the Environment and Life.

[62] Haraguchi H. (2004). Metallomics as integrated biometal science. J. Anal. At. Spectrom..

[63] Maret W. (2022). The quintessence of metallomics: A harbinger of a different life science based on the periodic table of the bioelements. Metallomics.

[64] Wohlgemuth R. (2021). Bio-based resources, bioprocesses and bioproducts in value creation architectures for bioeconomy markets and beyond—What really matters. Bioeconomy J..

[65] Winkler C.K., Schrittwieser J.H., Kroutil W. (2021). Power of biocatalysis for organic synthesis. ACS Cent. Sci..

[66] Wender P.A., Miller B.L. (2009). Synthesis at the molecular frontier. Nature.

[67] Schrittwieser J.H., Velikogne S., Hall M., Kroutil W. (2018). Artificial biocatalytic linear cascades for preparation of organic molecules. Chem. Rev..

[68] Zetzsche L.E., Chakrabarty S., Narayan A.R. (2022). The transformative power of biocatalysis in convergent synthesis. J. Am. Chem. Soc..

[69] Gerry C.J., Schreiber S.L. (2020). Recent achievements and current trajectories of diversity-oriented synthesis. Curr. Opin. Chem. Biol..

[70] Wohlgemuth R. (2022). Selective biocatalytic defunctionalization of raw materials. ChemSusChem.

[71] Reetz M.T., Qu G., Sun Z. (2024). Engineered enzymes for the synthesis of pharmaceuticals and other high-value products. Nat. Synth..

[72] Hönig M., Sondermann P., Turner N.J., Carreira E.M. (2017). Enantioselective Chemo- and Biocatalysis: Partners in Retrosynthesis. Angew. Chem. Int. Ed..

[73] de Souza R.O.M.A., Miranda L.S.M., Bornscheuer U.T. (2017). A Retrosynthesis Approach for Biocatalysis in Organic Synthesis. Chem.-Eur. J..

[74] Wohlgemuth R. (2023). Route selection and reaction engineering for sustainable metabolite synthesis. React. Chem. Eng..

[75] Simić S., Zukić E., Schmermund L., Faber K., Winkler C.K., Kroutil W. (2021). Shortening synthetic routes to small molecule active pharmaceutical ingredients employing biocatalytic methods. Chem. Rev..

[76] Wohlgemuth R. (2021). Biocatalysis–Key enabling tools from biocatalytic one-step and multi-step reactions to biocatalytic total syn- thesis. New Biotechnol..

[77] Kissman E.N., Sosa M.B., Millar D.C., Koleski E.J., Thevasundaram K., Chang M.C.Y. (2024). Expanding chemistry through in vitro and in vivo biocatalysis. Nature.

[78] Thibodeaux C.J., Melançon C.E., Liu H.-w. (2007). Unusual sugar biosynthesis and natural product glycodiversification. Nature.

[79] Wlodek A., Kendrew S.G., Coates N.J., Hold A., Pogwizd J., Rudder S., Sheehan L.S., Higginbotham S.J., Stanley-Smith A.E., Warneck T. (2017). Diversity oriented biosynthesis via accelerated evolution of modular gene clusters. Nat. Commun..

[80] Wittmann B.J., Knight A.M., Hofstra J.L., Reisman S.E., Kan S.B.J., Arnold F.H. (2020). Diversity-Oriented Enzymatic Synthesis of Cyclopropane Building Blocks. ACS Catal..

[81] Erb T.J., Zarzycki J. (2018). A short history of RubisCO: The rise and fall (?) of Nature’s predominant CO2 fixing enzyme. Curr. Opin. Biotechnol..

[82] Luo S., Diehl C., He H., Bae Y., Klose M., Claus P., Socorro Cortina N., Alvarez Fernandez C., Schulz-Mirbach H., McLean R. (2023). Construction and modular implementation of the THETA cycle for synthetic CO_2_ fixation. Nat. Catal..

[83] Chen P.-R., Xia P.-F. (2024). Carbon recycling with synthetic CO_2_ fixation pathways. Curr. Opin. Biotechnol..

[84] McLean R., Schwander T., Diehl C., Cortina N.S., Paczia N., Zarzycki J., Erb T.J. (2023). Exploring alternative pathways for the *in vitro* establishment of the HOPAC cycle for synthetic CO_2_ fixation. Sci. Adv..

[85] Scheffen M., Marchal D.G., Beneyton T., Schuller S.K., Klose M., Diehl C., Lehmann J., Pfister P., Carrillo M., He H. (2021). A new-to-nature carboxylation module to improve natural and synthetic CO_2_ fixation. Nat. Catal..

[86] Schwander T., Schada von Borzyskowski L., Burgener S., Cortina N.S., Erb T.J. (2016). A synthetic pathway for the fixation of carbon dioxide in vitro. Science.

[87] Diehl C., Gerlinger P.D., Paczia N., Erb T.J. (2023). Synthetic anaplerotic modules for the direct synthesis of complex molecules from CO_2_. Nat. Chem. Biol..

[88] Cai T., Sun H., Qiao J., Zhu L., Zhang F., Zhang J., Tang Z., Wei X., Yang J., Yuan Q. (2021). Cell-free chemoenzymatic starch synthesis from carbon dioxide. Science.

[89] Vidal F., van der Marel E.R., Kerr R.W.F., McElroy C., Schroeder N., Mitchell C., Rosetto G., Chen T.T.D., Bailey R.M., Hepburn C. (2024). Designing a circular carbon and plastics economy for a sustainable future. Nature.

[90] Tournier V., Duquesne S., Guillamot F., Cramail H., Taton D., Marty A., André I. (2023). Enzymes’ power for plastics degradation. Chem. Rev..

[91] Tournier V., Topham C.M., Gilles A., David B., Folgoas C., Moya-Leclair E., Kamionka E., Desrousseaux M.-L., Texier H., Gavalda S. (2020). Enzymes’ Power for Plastics Degradation. Nature.

[92] Bell E.L., Rosetto G., Ingraham M.A., Ramirez K.J., Lincoln C., Clarke R.W., Gado J.E., Lilly J.L., Kucharzyk K.H., Erickson E. (2024). Natural diversity screening, assay development, and characterization of nylon-6 enzymatic depolymerization. Nat. Commun..

[93] Bayer T., Palm G.J., Berndt L., Meinert H., Branson Y., Schmidt L., Cziegler C., Somvilla I., Zurr C., Graf L.G. (2024). Structural Elucidation of a Metagenomic Urethanase and Its Engineering Towards Enhanced Hydrolysis Profiles. Angew. Chem. Int. Ed..

[94] Lima P.J.M., da Silva R.M., Neto C.A.C.G., Gomes e Silva N.C., Souza J.E.D.S., Nunes Y.L., Sousa dos Santos J.C. (2022). An overview on the conversion of glycerol to value-added industrial products via chemical and biochemical routes. Biotechnol. Appl. Biochem..

[95] Li Z., Yan J., Sun J., Xu P., Ma C., Gao C. (2018). Production of value-added chemicals from glycerol using in vitro enzymatic cascades. Commun. Chem..

[96] Moklis M.H., Cheng S., Cross J.S. (2023). Current and future trends for crude glycerol upgrading to high value-added products. Sustainability.

[97] Guajardo N., Bernal C., Wilson L., Cabrera Z. (2015). Selectivity of *R*-α-monobenzoate glycerol synthesis catalyzed by *Candida antarctica* lipase B immobilized on heterofunctional supports. Proc. Biochem..

[98] Richter N., Neumann M., Liese A., Wohlgemuth R., Eggert T., Hummel W. (2009). Characterisation of a Recombinant NADP-Dependent Glycerol Dehydrogenase from *Gluconobacter oxydans* and its Application in the Production of L-Glyceraldehyde. ChemBioChem.

[99] Richter N., Neumann M., Liese A., Wohlgemuth R., Weckbecker A., Eggert T., Hummel W. (2010). Characterization of a whole-cell catalyst co-expressing glycerol dehydrogenase and glucose dehydrogenase and its application in the synthesis of L-glyceraldehyde. Biotechnol. Bioeng..

[100] Habe H., Shimada Y., Yakushi T., Hattori H., Ano Y., Fukuoka T., Kitamoto D., Itagaki M., Watanabe K., Yanagishita H. (2009). Microbial Production of Glyceric Acid, an Organic Acid That Can Be Mass Produced from Glycerol. Appl. Env. Microbiol..

[101] Zhang C., Chen Q., Fan F., Tang J., Zhan T., Wang H., Zhang X. (2021). Directed evolution of alditol oxidase for the production of optically pure D-glycerate from glycerol in the engineered *Escherichia coli*. J. Ind. Microbiol. Biotechnol..

[102] Rios-Mercadillo V.M., Whitesides G.M. (1979). Enzymic synthesis of *sn*-glycerol 3-phosphate. J. Am. Chem. Soc..

[103] Ragauskas A.J., Williams C.K., Davison B.H., Britovsek G., Cairney J., Eckert C.A., Frederick W.J., Hallett J.P., Leak D.J., Liotta C.L. (2006). The path forward for biofuels and biomaterials. Science.

[104] Sheldon R.A. (2020). Biocatalysis and biomass conversion: Enabling a circular economy. Philos. Trans. R. Soc. A..

[105] Hoff B., Plassmeier J., Blankschien M., Letzel A.C., Kourtz L., Schröder H., Koch W., Zelder O. (2021). Unlocking Nature’s Biosynthetic Power—Metabolic Engineering for the Fermentative Production of Chemicals. Angew. Chem. Int. Ed..

[106] Clomburg J.M., Crumbley A.M., Gonzalez R. (2017). Industrial biomanufacturing: The future of chemical production. Science.

[107] Brun N., Hesemann P., Esposito D. (2017). Expanding the biomass derived chemical space. Chem. Sci..

[108] Sheldon R.A. (2024). Green carbon and the chemical industry of the future. Philos. Trans. R. Soc. A.

[109] Reichstein T., Grüssner A., Oppenauer R. (1933). Synthesis of *d*-and *l*-ascorbic acid (vitamin C). Nature.

[110] Reichstein T., Grüssner A. (1934). Eine ergiebige Synthese der L-ascorbinsäure (C-vitamin). Helv. Chim. Acta.

[111] Pappenberger G., Hohmann H.P., Zorn H., Czermak P. (2014). Industrial production of L-ascorbic acid (vitamin C) and D-isoascorbic acid. Biotechnology of Food and Feed Additives.

[112] Wang Y., Liu L., Jin Z., Zhang D. (2021). Microbial cell factories for green production of vitamins. Front. Bioeng. Biotechnol..

[113] Scott A.I. (2003). Discovering nature’s diverse pathways to vitamin B_12_: A 35-year odyssey. J. Org. Chem..

[114] Kang Q., Fang H., Xiang M., Xiao K., Jiang P., You C., Lee S.Y., Zhang D. (2023). A synthetic cell-free 36-enzyme reaction system for vitamin B_12_ production. Nat. Commun..

[115] Martens J.-H., Barg H., Warren M.J., Jahn D. (2002). Microbial production of vitamin B_12_. Appl. Microbiol. Biotechnol..

[116] Jani P., Emmert J., Wohlgemuth R. (2008). Process analysis of macrotetrolide biosynthesis during fermentation by means of direct infusion LC-MS. Biotechnol. J..

[117] Smanski M., Zhou H., Claesen J., Shen B., Fischbach M.A., Voigt C.A. (2016). Synthetic biology to access and expand nature’s chemical diversity. Nat. Rev. Microbiol..

[118] Nielsen J., Keasling J.D. (2016). Engineering cellular metabolism. Cell.

[119] Lee S.Y., Kim H.U., Chae T.U., Cho J.S., Kim J.W., Shin J.H., Kim D.I., Ko Y.-S., Jang W.D., Jang Y.-S. (2019). A comprehensive metabolic map for production of bio-based chemicals. Nat. Catal..

[120] Sheldon R.A., Brady D. (2022). Green chemistry, biocatalysis, and the chemical industry of the future. ChemSusChem.

[121] Aggarwal N., Pham H.L., Ranjan B., Saini M., Liang Y., Hossain G.S., Ling H., Foo J.L., Chang M.W. (2024). Microbial engineering strategies to utilize waste feedstock for sustainable bioproduction. Nat. Rev. Bioeng..

[122] Cho E.J., Trinh L.T.P., Song Y., Lee Y.G., Bae H.-J. (2020). Bioconversion of biomass waste into high value chemicals. Bioresour. Technol..

[123] Wohlgemuth R., Littlechild J. (2022). Complexity reduction and opportunities in the design, integration and intensification of biocata-lytic processes for metabolite synthesis. Front. Bioeng. Biotechnol..

[124] Sun H., Zhang H., Ang E.L., Zhao H. (2018). Biocatalysis for the synthesis of pharmaceuticals and pharmaceutical intermediates. Bioorg. Med. Chem..

[125] Lewis R.D., France S.P., Martinez C.A. (2023). Emerging technologies for biocatalysis in the pharmaceutical industry. ACS Catal..

[126] Naik K., Dheeraj S., Jeevani K., Saravanan T. (2024). Evaluating Multienzyme Cascade Routes for Pharmaceutically Relevant Mole-cules. Eur. J. Org. Chem..

[127] Etit D., Meramo S., Ögmundarson Ó., Jensen M.K., Sukumara S. (2024). Can biotechnology lead the way toward a sustainable pharmaceutical industry?. Curr. Opin. Biotechnol..

[128] Wegman M.A., Janssen M.H.A., van Rantwijk F., Sheldon R.A. (2001). Towards Biocatalytic Synthesis of β-Lactam Antibiotics. Adv. Synth. Catal..

[129] Huffman M.A., Fryszkowska A., Alvizo O., Borra-Garske M., Campos K.R., Canada K.A., Devine P.N., Duan D., Forstater J.H., Grosser S.T. (2019). Design of an in vitro biocatalytic cascade for the manufacture of islatravir. Science.

[130] Rummelt S.M., Qi J., Chen Y., Dropinski J.F., Hughes G., Kuethe J.T., Li D., Maloney K.M., Margelefsky E., Mathew R. (2021). Development of an Efficient Route to 2-Ethynylglycerol for the Synthesis of Islatravir. ChemRxiv.

[131] Shaw M.H., Fryszkowska A., Alvizo O., Attadgie I., Borra-Garske M., Devine P.N., Duan D., Grosser S.T., Forstater J.H., Hughes G.J. (2023). Development of a Biocatalytic Aerobic Oxidation for the Manufacturing Route to Islatravir. ChemRxiv.

[132] McIntosh J.A., Benkovics T., Silverman S.M., Huffman M.A., Kong J., Maligres P.E., Itoh T., Yang H., Verma D., Pan W. (2021). Engineered ribosyl-1-kinase enables concise synthesis of molnupiravir, an antiviral for COVID-19. ACS Cent. Sci..

[133] Avci F.Y., DeAngelis P.L., Liu J., Linhardt R.J. (2007). Enzymatic Synthesis of Glycosaminoglycans: Improving on Nature. Front. Mod. Carbohydr. Chem..

[134] Zheng J., Lin X.J., Xu H., Sohail M., Chen L.A., Zhang X. (2024). Enzyme-mediated green synthesis of glycosaminoglycans and catalytic process intensification. Biotechnol. Adv..

[135] Gottschalk J., Elling L. (2021). Current state on the enzymatic synthesis of glycosaminoglycans. Curr. Opin. Chem. Biol..

[136] Gottschalk J., Aßmann M., Kuballa J., Elling L. (2022). Repetitive Synthesis of High-Molecular-Weight Hyaluronic Acid with Immobilized Enzyme Cascades. ChemSusChem.

[137] Vera C., Illanes A., Guerrero C. (2021). Enzymatic production of prebiotic oligosaccharides. Curr. Opin. Food Sci..

[138] Castejón N., Señoráns F.J. (2020). Enzymatic modification to produce health-promoting lipids from fish oil, algae and other new omega-3 sources: A review. New Biotechnol..

[139] Hua Z., Wu Q., Yang Y., Liu S., Tchuere J.G., Zhao D., Fang Y. (2024). Essential roles of ellagic acid-to-urolithin converting bacteria in human health and health food industry: An updated review. Trends Food Sci. Technol..

[140] Eichhorn E., Locher E., Guillemer S., Wahler D., Fourage L., Schilling B. (2018). Biocatalytic process for (−)-ambrox production using squalene hopene cyclase. Adv. Synth. Catal..

[141] Eichhorn E., Schroeder F. (2023). From Ambergris to (−)-Ambrox: Chemistry meets biocatalysis for sustainable (−)-Ambrox production. J. Agric. Food Chem..

[142] Heath R.S., Birmingham W.R., Thompson M.P., Taglieber A., Daviet L., Turner N.J. (2019). An engineered alcohol oxidase for the oxidation of primary alcohols. ChemBioChem.

[143] Hall M. (2021). Enzymatic strategies for asymmetric synthesis. RSC Chem. Biol..

[144] Wohlgemuth R., De Gonzalo G., Alcántara A.R. (2024). Industrial asymmetric biocatalysis. Biocatalysis in Asymmetric Synthesis.

[145] Rowbotham J.S., Ramirez M.A., Lenz O., Reeve H.A., Vincent K.A. (2020). Bringing biocatalytic deuteration into the toolbox of asymmetric isotopic labelling techniques. Nat. Commun..

[146] Mugford P.F., Wagner U.G., Jiang Y., Faber K., Kazlauskas R.J. (2008). Enantiocomplementary enzymes: Classification, molecular basis for their enantiopreference, and prospects for mirror-image biotransformations. Angew. Chem. Int. Ed..

[147] Messiha H.L., Scrutton N.S., Leys D. (2023). High-Titer Bio-Styrene Production Afforded by Whole-Cell Cascade Biotransformation. ChemCatChem.

[148] Hinzmann A., Stricker M., Gröger H. (2020). Chemoenzymatic Cascades toward Aliphatic Nitriles Starting from Biorenewable Feedstocks. ACS Sustain. Chem. Eng..

[149] Hinzmann A., Glinski S., Worm M., Gróger H. (2019). Enzymatic synthesis of aliphatic nitriles at a substrate loading of up to 1.4 kg/L: A biocatalytic record achieved with a heme protein. J. Org. Chem..

[150] Straathof A.J.J. (2014). Transformation of Biomass into Commodity Chemicals Using Enzymes or Cells. Chem. Rev..

[151] Sachs J.D., Schmidt-Traub G., Mazzucato M., Messner D., Nakicenovic N., Rockström J. (2019). Six Transformations to achieve the Sustainable Development Goals. Nat. Sustain..

[152] Liu Z., Shi S., Ji Y., Wang K., Tan T., Nielsen J. (2023). Opportunities of CO_2_-based biorefineries for production of fuels and chemicals. Green Carbon.

[153] Liew F.E., Nogle R., Abdalla T., Rasor B.J., Canter C., Jensen R.O., Wang L., Strutz J., Chirania P., De Tissera S. (2022). Carbon-negative production of acetone and isopropanol by gas fermentation at industrial pilot scale. Nat. Biotechnol..

[154] Gardossi L., Poulsen P.B., Ballesteros A., Hult K., Švedas V.K., Vasić-Rački Đ., Carrea G., Magnusson A., Schmid A., Wohlgemuth R. (2010). Guidelines for reporting of biocatalytic reactions. Trends Biotechnol..

[155] Swainston N., Baici A., Bakker B.M., Cornish-Bowden A., Fitzpatrick P.F., Halling P., Leyh T.S., O’Donovan C., Raushel F.M., Reschel U. (2018). STRENDA DB: Enabling the validation and sharing of enzyme kinetics data. FEBS J..

[156] Aguilar A., Twardowski T., Wohlgemuth R. (2019). Bioeconomy for sustainable development. Biotechnol. J..

[157] Wohlgemuth R., Twardowski T., Aguilar A. (2021). Bioeconomy moving forward step by step–A global journey. New Biotechnol..

